# Magnet Recognition, Leadership Responsiveness, and Nurse Moral Distress: A Multilevel Mediation Analysis

**DOI:** 10.1111/jnu.70144

**Published:** 2026-10-13

**Authors:** Hyunmin Yu, Daniel McNeish, Celsea Tibbitt, Christin Iroegbu, Matthew D. McHugh

**Affiliations:** ^1^ Center for Health Outcomes and Policy Research, School of Nursing University of Pennsylvania Philadelphia Pennsylvania USA; ^2^ Leonard Davis Institute of Health Economics University of Pennsylvania Philadelphia Pennsylvania USA; ^3^ Arizona State University Tempe Arizona USA; ^4^ Towson University Towson Maryland USA

**Keywords:** hospitals, leadership, Magnet Recognition Program, moral distress, nurses

## Abstract

**Purpose:**

Moral distress is a pervasive challenge in nursing and has been associated with burnout, job dissatisfaction, and turnover. Although Magnet Recognition has been associated with more favorable nursing practice environments and nurse outcomes, little is known about the association between Magnet status and nurses' experiences of moral distress or the role of leadership responsiveness in this association. This study examined whether management responsiveness to patient care concerns accounted for part of the association between hospital Magnet status and nurse moral distress.

**Design:**

This was a cross‐sectional observational study.

**Methods:**

Data from the 2024 Penn Nurses4All Survey and the 2023 American Hospital Association Annual Survey were linked. The analytic sample included 18,678 direct care nurses from 1829 hospitals across 10 US states. The dependent variable was nurse moral distress, the independent variable was hospital Magnet status, and the mediator was nurse‐reported management responsiveness to patient care concerns. A 2‐1‐1 multilevel mediation model using multilevel structural equation modeling was estimated to distinguish within‐ and between‐hospital associations while accounting for the clustering of nurses within hospitals. Models adjusted for nurse‐ and hospital‐level characteristics.

**Results:**

Nurses in Magnet hospitals reported lower levels of moral distress and more favorable assessments of management responsiveness than nurses in non‐Magnet hospitals. At the between‐hospital level, Magnet status was associated with higher management responsiveness (unstandardized coefficient *b* = 0.18, 95% CI = 0.15 to 0.21, *p* < 0.001), and higher management responsiveness was associated with lower moral distress (*b* = −0.31, 95% CI = −0.39 to −0.23, *p* < 0.001). Greater management responsiveness was also associated with lower moral distress at the within‐hospital level (*b* = −0.34, 95% CI = −0.35 to −0.33, *p* < 0.001). The direct between‐hospital association between Magnet status and moral distress remained statistically significant after accounting for management responsiveness (*b* = −0.05, 95% CI = −0.08 to −0.01, *p* = 0.011). The between‐hospital indirect association between Magnet status and moral distress involving management responsiveness was statistically significant (*b* = −0.06, Monte Carlo 95% CI = −0.08 to −0.04, *p* < 0.001). Similar findings were observed using administrative responsiveness to nurse concerns.

**Conclusions:**

Nurses working in Magnet hospitals reported lower levels of moral distress and more favorable assessments of management responsiveness than nurses working in non‐Magnet hospitals. Management responsiveness statistically accounted for part of the observed between‐hospital association between Magnet status and moral distress.

**Clinical Relevance:**

Leadership responsiveness to nurses' patient care concerns may represent an important and potentially modifiable organizational factor associated with moral distress. Organizational strategies to strengthen management responsiveness and nurses' ability to raise patient care concerns warrant further evaluation as approaches to supporting nurse well‐being.

## 1 Introduction

Moral distress is a pervasive challenge in nursing (Alimoradi et al. 2023; Lake et al. 2022; Salari et al. 2022). It occurs when nurses are unable to act in accordance with what they believe is ethically appropriate for their patients because of organizational, interpersonal, or resource‐related constraints (Morley et al. 2019). Moral distress has been linked to numerous adverse outcomes, including burnout, job dissatisfaction, and turnover (Eche et al. 2023; Karakachian and Colbert 2019). Given ongoing workforce shortages, retention challenges, and the increasing complexity of healthcare delivery, identifying organizational factors associated with moral distress remains a critical priority for nursing research and practice.

Previous research has identified a variety of factors associated with moral distress, including individual nurse characteristics (e.g., years of experience and personal traits), patient acuity, and ethical climate (Atashzadeh‐Shoorideh et al. 2021; Lamoureux et al. 2024). Although much of this research has focused on nurse‐level and patient‐level contributors to moral distress, growing evidence suggests that organizational factors also play an important role in shaping nurses' experiences of moral distress. Organizational environments, including staffing adequacy, resource availability, and leadership support (Lake, Smith, et al. 2024), influence nurses' ability to advocate for patients, communicate concerns, participate in decision‐making, and obtain support when ethical challenges arise (Booth and Robinson 2025). Consequently, organizational characteristics that facilitate responsiveness to nurses' concerns may represent important but understudied factors associated with moral distress.

Management responsiveness to patient care concerns raised by direct care nurses may represent an important organizational factor associated with nurses' experiences of moral distress, as leadership plays a critical role in shaping workplace culture and ensuring the availability of resources needed to deliver high‐quality patient care (McSherry et al. 2012). A recent scoping review suggests that nurse leaders may play a key role in supporting nursing staff through ethically challenging and emotionally distressing situations (Bolton et al. 2025). By demonstrating authentic leadership, ethical decision‐making, and advocacy for staff, nurse leaders can foster supportive and psychologically safe work environments.

Existing literature on the management of moral distress further emphasizes the importance of organizational and leadership responses. Strategies described for addressing moral distress include creating opportunities for ethical reflection and discussion, strengthening interdisciplinary communication, providing access to ethics resources and support, and fostering organizational cultures in which nurses feel able to raise concerns and participate in decision‐making (Morley et al. 2021). Leadership support is particularly relevant because nurses' ability to respond to ethically challenging situations may depend not only on their individual coping resources but also on whether organizational leaders acknowledge concerns and facilitate appropriate action (Bolton et al. 2025). However, much of this literature has focused on strategies for responding to moral distress or on general characteristics of ethical and supportive work environments. Less is known about nurses' assessments of whether management will respond to patient care concerns they identify and how such responsiveness is associated with moral distress across hospitals.

These leadership principles are closely aligned with the Magnet Recognition Program. Magnet Recognition (Magnet), administered by the American Nurses Credentialing Center (ANCC), is widely recognized as a marker of nursing excellence and supportive professional practice environments (American Nurses Credentialing Center 2025). Previous research examining organizational factors associated with the more favorable nurse and patient outcomes observed in Magnet hospitals has considered several factors, including stronger nursing practice environments, inclusive organizational culture, transformational leadership, structural empowerment, professional autonomy, shared governance, and greater nurse participation in organizational decision‐making (Fischer and Nichols 2019; Gonzalez et al. 2015; Kutney‐Lee et al. 2015; Yu et al. 2025). These factors characterize important organizational structures and conditions that support professional nursing practice.

Management responsiveness is conceptually related to, but distinct from, the broader organizational characteristics emphasized by Magnet. Whereas shared governance, structural empowerment, and participation in decision‐making provide organizational structures through which nurses can exercise professional voice and raise concerns, management responsiveness reflects a more proximal organizational process of how management responds when nurses identify problems in patient care. Thus, management responsiveness may represent one process by which the broader organizational structures associated with Magnet status are reflected in nurses' day‐to‐day experiences.

### 1.1 Theoretical Framework

This study was informed by the Structure‐Process‐Outcome (SPO) framework (Donabedian 2002), which conceptualizes organizational structures as the context within which care processes occur and outcomes are experienced. Within this framework, Magnet status was conceptualized as a hospital‐level structural characteristic reflecting a broader organizational context that emphasizes nursing leadership, structural empowerment, professional practice, and nurse participation in decision‐making. Management responsiveness to patient care concerns was conceptualized as a process‐related factor reflecting nurses' assessments of how management responds when they identify problems in patient care. Although management responsiveness is closely related to the leadership and empowerment principles embedded within Magnet, it captures nurses' perceptions of how these organizational principles are enacted in response to specific patient care concerns, rather than the presence of the broader organizational structures themselves. Moral distress was conceptualized as a nurse outcome associated with nurse well‐being, retention, and quality of care.

Consistent with the SPO framework, Magnet status may be associated with more favorable management responsiveness given the program's emphasis on organizational structures that support nurse voice, shared decision‐making, and leadership engagement. Greater management responsiveness may, in turn, be associated with lower moral distress because nurses who perceive management as responsive to patient care concerns may feel better supported in advocating for patients and addressing barriers to ethically appropriate care. Conversely, perceived management unresponsiveness may be associated with greater moral distress when organizational barriers constrain nurses' ability to provide the care they believe is ethically appropriate.

Therefore, the purpose of this study was to examine whether management responsiveness to patient care concerns accounts for part of the association between hospital Magnet status and nurse moral distress. We hypothesized that: (1) nurses working in Magnet hospitals would report lower levels of moral distress and higher levels of management responsiveness than nurses working in non‐Magnet hospitals; (2) higher levels of management responsiveness would be associated with lower levels of moral distress; and (3) management responsiveness would partially account for the association between Magnet status and nurse moral distress.

## 2 Methods

### 2.1 Study Design and Data Sources

This cross‐sectional observational study linked two data sources using a shared hospital identifier: the 2024 Penn Nurses4All Survey and the 2023 American Hospital Association (AHA) Annual Survey. The Nurses4All Survey is a large multistate survey of registered nurses that collects information on nurses' work environments and employment experiences (Muir et al. 2025; Yu, Connell, et al. 2026). The survey examined nurse practice settings and job outcomes across 10 US states (California, Florida, Illinois, Louisiana, New Jersey, New Mexico, New York, Oregon, Pennsylvania, and Washington). Between December 2023 and March 2024, survey invitations were emailed to all actively licensed registered nurses in nine states using state licensure databases. In Pennsylvania, where only mailing addresses were available, a random 30% sample of licensed nurses received the survey (Muir et al. 2026).

Consistent with previous studies (Lasater et al. 2021, 2019), a modified Dillman survey approach was used (Dillman et al. 2014), with reminder contacts sent periodically to nonrespondents. Prior validation studies using intensive follow‐up of nurses who did not initially respond found minimal evidence of nonresponse bias (Lasater et al. 2019). The sample included nurses working across diverse healthcare settings, including hospitals, long‐term care facilities, schools, and primary care practices, as well as retired or unemployed nurses maintaining active licensure. Hospital‐level characteristics were obtained from the 2023 AHA Annual Survey (American Hospital Association 2023), and Magnet status was identified using the ANCC's public database (American Nurses Credentialing Center 2026).

### 2.2 Study Sample

To evaluate the associations among Magnet status, nurses' assessments of management responsiveness to patient care concerns, and moral distress, we included nurses who: (1) reported working in hospitals and identified their hospital by name, given that Magnet designation primarily applies to hospital settings; (2) provided direct patient care; and (3) completed measures related to management responsiveness and moral distress. Nurses who did not work in hospital settings, did not provide direct patient care, or did not provide data on moral distress and management responsiveness were excluded from the analytic sample. The study sample selection process, including the numbers of nurses screened, excluded at each stage, and included in the final analytic sample, is presented in Figure 1. The final study sample included 18,678 direct care nurses employed across 1829 hospitals in 10 U.S. states. The median number of nurses per hospital was 5 (range, 1–161).

**FIGURE 1:**
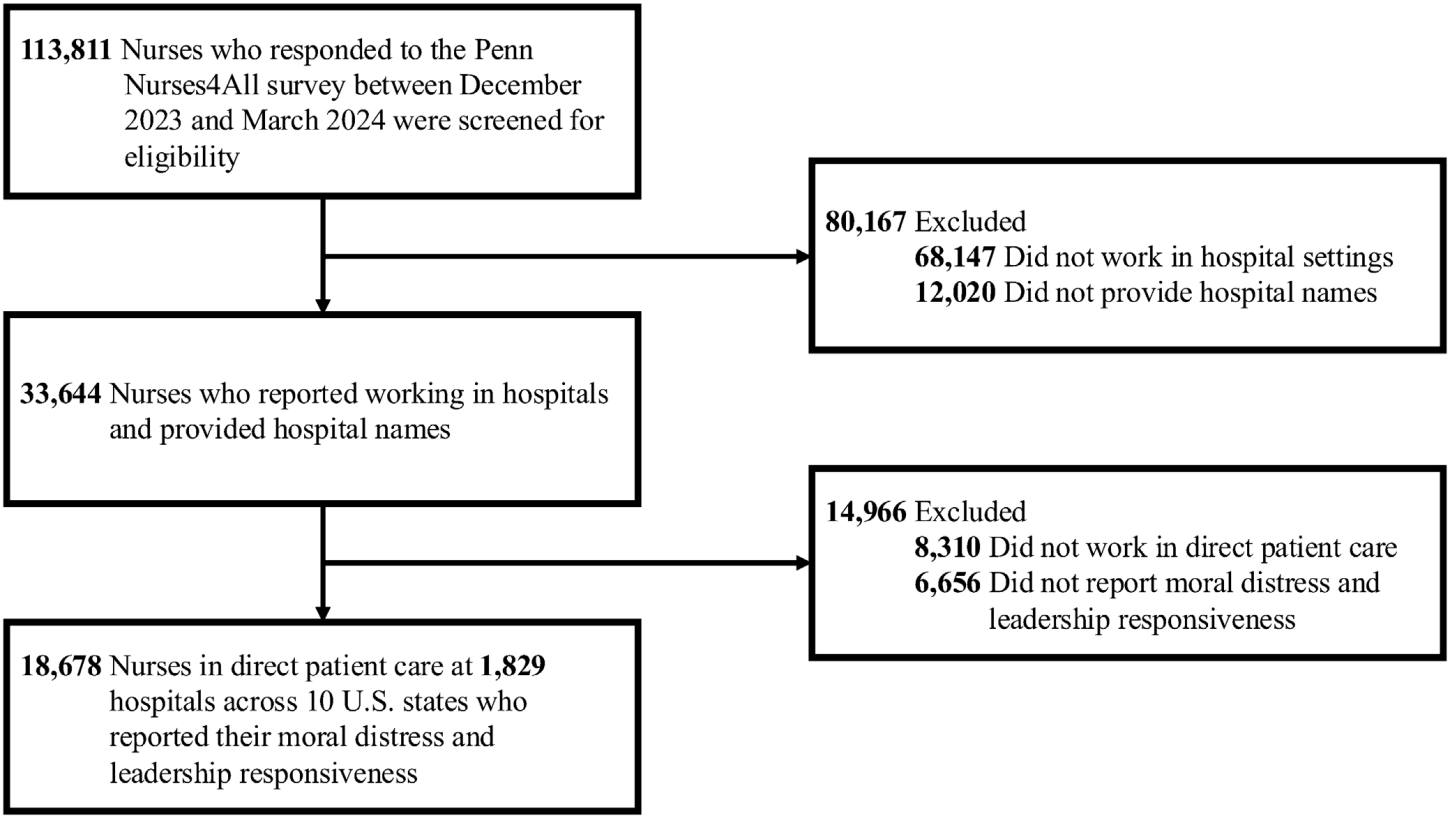
Study Sample Selection Flow Diagram. This figure presents the selection of the analytic sample from respondents to the Penn Nurses4All survey conducted between December 2023 and March 2024. The final analytic sample included 18,678 direct care nurses from 1829 hospitals across 10 US states who reported moral distress and leadership responsiveness.

### 2.3 Dependent, Independent, and Mediating Variables

#### 2.3.1 Dependent Variable: Moral Distress

Moral distress was measured using a single survey item based on Jameton's widely cited definition of moral distress (Jameton 1984). Respondents were asked to indicate the extent of moral distress they experienced in their work: “Moral distress arises when constraints prevent us from doing the right thing for patients. How much moral distress do you experience related to your work?” Responses were recorded on a 4‐point Likert scale ranging from 1 (severe) to 4 (none). To facilitate interpretation, scores were reverse coded so that higher values reflected higher levels of moral distress.

#### 2.3.2 Independent Variable: Magnet Status

The independent variable was a binary indicator of hospital Magnet status. Given that Nurses4All data collection occurred from December 2023 through March 2024, we examined changes in Magnet designation among hospitals in the analytic sample during this period. Three (0.2%) hospitals received Magnet designation between January and March 2024; no hospitals received Magnet designation in December 2023. These hospitals were classified as Magnet hospitals. Hospitals with an active Magnet designation during the study period in 2024 were coded as 1, whereas hospitals without a Magnet designation were coded as 0. Magnet designation status was identified using the ANCC public database (American Nurses Credentialing Center 2026).

#### 2.3.3 Mediating Variable: Management Responsiveness to Patient Care Concerns

As the primary mediator, management responsiveness to patient care concerns was measured using a single‐item question asking nurses, “How confident are you that management will act to resolve problems in patient care that nurses identify?” Response options ranged from 1 (very confident) to 4 (not at all confident). Responses were reverse coded so that higher scores indicated a more favorable assessment of management responsiveness to patient care concerns.

As a sensitivity analysis, we examined a conceptually related measure of administrative responsiveness derived from the Practice Environment Scale‐5 (PES‐5) (Lake, Gil, et al. 2024). Nurses were asked to indicate their level of agreement with the statement, “Administration that listens and responds to nurse concerns.” Responses were recorded on a 4‐point Likert scale ranging from 1 (strongly agree) to 4 (strongly disagree). Responses were reverse coded such that higher scores reflected a more favorable assessment of administrative responsiveness to nurse concerns. Consistency of findings across both measures was used to evaluate the robustness of the primary mediation results.

### 2.4 Covariates

#### 2.4.1 Nurse‐Level (Level 1)

We adjusted for several nurse‐level (Level 1) characteristics that have been associated with moral distress, including age (Dodek et al. 2016), sex (O'Connell 2015), educational attainment (Lawrence 2011), employment status (Lazzarin et al. 2012), and practice setting (Alimoradi et al. 2023). Age was analyzed as a continuous variable. Sex was coded as 0 = female and 1 = male. The original categories for highest nursing education included licensed practical nurse training, hospital diploma, associate degree, bachelor's degree, master's degree, and doctoral degree. For analysis, educational attainment was dichotomized as 0 = less than a bachelor's degree and 1 = bachelor's degree or higher. Employment status was classified as 0 = full‐time and 1 = part‐time. Given prior evidence demonstrating elevated levels of moral distress among nurses working in psychiatric units and critical care settings (Alimoradi et al. 2023), practice setting was categorized as 1 = psychiatric or critical care settings and 0 = all other settings.

To distinguish within‐hospital differences in nurse characteristics from differences in nurse composition across hospitals, all nurse‐level covariates were decomposed into within‐ and between‐hospital components (Enders and Tofighi 2007). For each nurse characteristic, the individual‐level value was group‐mean‐centered within hospitals for inclusion at Level 1. Corresponding hospital‐level means were calculated for age, and hospital‐level proportions were calculated for the binary characteristics (sex, educational attainment, employment status, and practice setting) for inclusion at Level 2.

#### 2.4.2 Hospital‐Level (Level 2)

We also adjusted for hospital‐level (Level 2) characteristics previously associated with nurse job outcomes, including teaching status, hospital size, specialized service capacity (Aiken et al. 2002), and ownership (Hansen et al. 2009). Teaching status was determined using the ratio of resident physicians and fellows to total hospital beds, a measure considered more accurate for assessing teaching intensity than university affiliation or professional memberships (Ayanian et al. 1998). Hospitals were categorized as 0 = non‐teaching and 1 = teaching, including minor teaching (resident‐to‐bed ratio ≤ 1:4) and major teaching (resident‐to‐bed ratio > 1:4). Hospital size was classified based on bed capacity as 0 = more than 250 beds and 1 = 250 beds or fewer (Aiken et al. 2002). Hospitals were categorized as 1 = high specialized service capacity if they provided advanced tertiary services (i.e., open‐heart surgery or major organ transplantation) (Hartz et al. 1989). Hospital ownership was categorized according to governing structure as 0 = not‐for‐profit and 1 = for‐profit.

### 2.5 Statistical Analysis

All analyses were conducted using R (version 4.5.2). We began with descriptive analyses of nurse‐ and hospital‐level characteristics. We then conducted a 2–1‐1 multilevel mediation analysis, in which the independent variable is measured at Level 2 (hospital level) and both the mediator and outcome are measured at Level 1 (nurse level), using multilevel structural equation modeling (MSEM) (Preacher et al. 2011, 2010) to examine whether nurses' assessments of management responsiveness to patient care concerns accounted for part of the association between hospital Magnet status and nurse moral distress while accounting for the nested structure of the data (i.e., nurses clustered within hospitals) (Figure 2).

**FIGURE 2:**
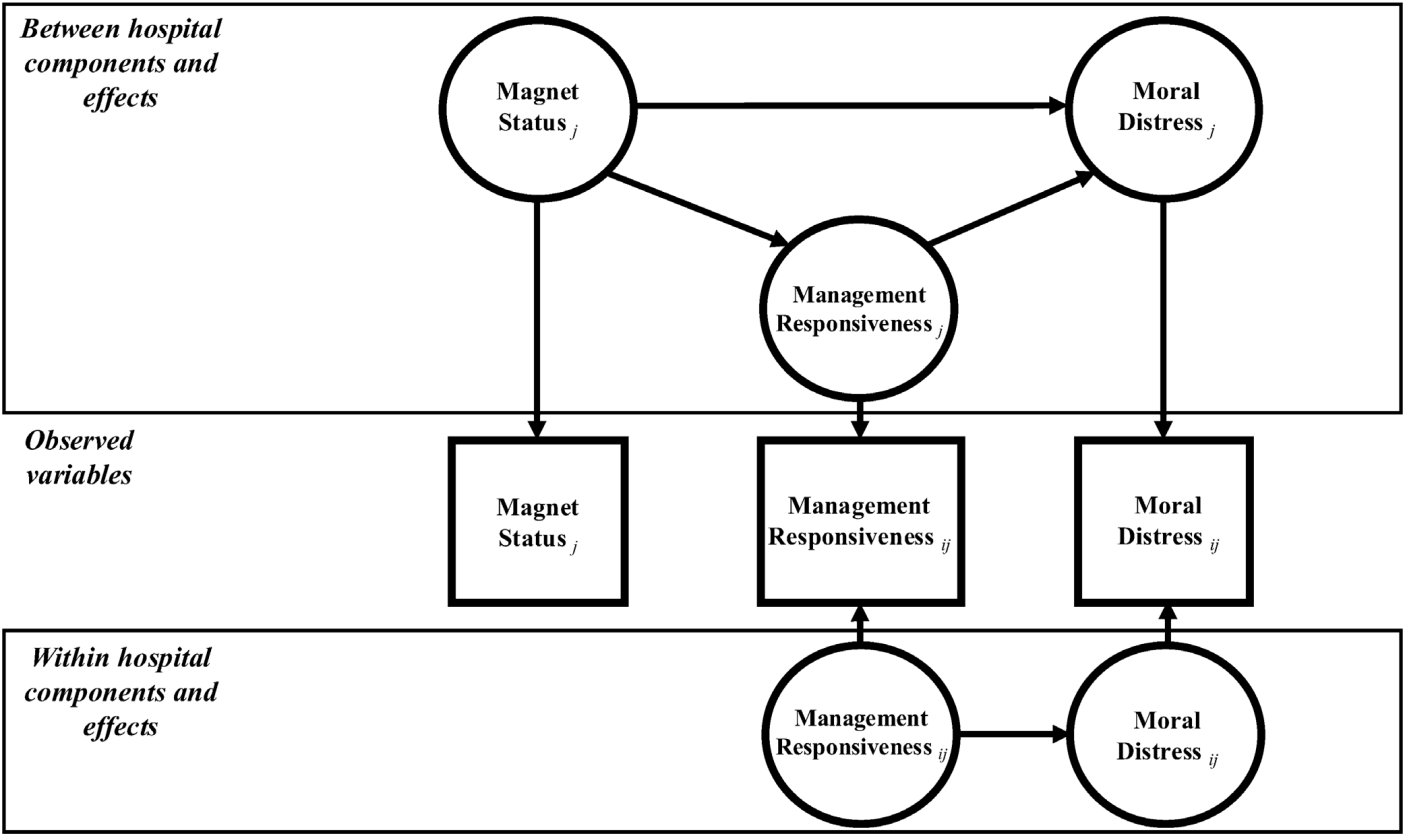
Hypothesized 2–1‐1 Multilevel Mediation Model. This figure presents the conceptual 2–1‐1 multilevel mediation model examining management responsiveness to patient care concerns as a mediator of the association between Magnet status and nurse moral distress. Magnet status is a hospital‐level predictor (Level 2), whereas management responsiveness and moral distress are measured at the nurse level (Level 1) and decomposed into between‐hospital and within‐hospital components. At the between‐hospital level, the model estimates the association between Magnet status and management responsiveness (*a*_B_), the association between management responsiveness and moral distress (*b*_B_), and the direct association between Magnet status and moral distress (*c'*_B_). The indirect association is estimated at the between‐hospital level as *a*_B_ × *b*_B_. At the within‐hospital level, the model estimates the association between management responsiveness and moral distress (*b*_W_). The model accounts for the clustering of nurses within hospitals.

Consistent with recommendations for multilevel mediation analyses (Preacher et al. 2011), MSEM was used to separate within‐ and between‐hospital associations and avoid conflating these sources of variation, which can bias estimates obtained from traditional multilevel mediation approaches. Specifically, Magnet status was modeled as a predictor of between‐hospital differences in management responsiveness (path *a*_B_), and management responsiveness was modeled as a predictor of moral distress at both the between‐hospital (path *b*_B_) and within‐hospital (path *b*_W_) levels. The direct between‐hospital association between Magnet status and moral distress was represented by path *c'*_B_. This approach allowed us to distinguish the within‐hospital association between management responsiveness and moral distress from the between‐hospital associations among Magnet status, management responsiveness, and moral distress.

MSEM was conducted using the lavaan package in R (Rosseel 2012). The model included a direct association between Magnet status and nurse moral distress (path *c'*_B_) and an indirect association involving management responsiveness (paths *a*_B_ and *b*_B_). The between‐hospital indirect association was estimated as the product of paths *a*_B_ and *b*_B_ (*a*_B_ × *b*_B_), and the total association was estimated as the sum of the direct and indirect associations. To appropriately account for the asymmetric sampling distribution of the indirect association in MSEM (Fang et al. 2019), Monte Carlo confidence intervals were estimated for the indirect association.

To account for differences in nurse composition across hospitals, nurse characteristics were decomposed into within‐ and between‐hospital components. Group‐mean‐centered age, sex, educational attainment, employment status, and practice setting were included as covariates at Level 1, while the corresponding hospital means or proportions were included as covariates at Level 2. Thus, the between‐hospital associations were adjusted for differences in the composition of nurses surveyed across hospitals. Level 2 models additionally adjusted for hospital teaching status, hospital size, specialized service capacity, and ownership. Because management responsiveness demonstrated moderate skewness, robust maximum likelihood estimation (MLR) was used to obtain standard errors and test statistics robust to non‐normality. Although management responsiveness and moral distress were ordinal, they were treated as continuous, consistent with recommendations supporting the use of robust maximum likelihood estimation for variables with four or more response categories and moderate departures from normality.

Overall, 83.8% of nurses had complete covariate data. Missingness was limited to covariates. Magnet status, moral distress, both leadership responsiveness measures, and practice setting had no missing values in the analytic sample. Among nurse‐level covariates, missingness was 5.6% for age, 4.2% for sex, 2.9% for educational attainment, and 0.1% for employment status. Among hospital‐level covariates, hospital size, teaching status, ownership, and specialized service capacity were missing for 320 of 1829 hospitals (17.5%). All 320 hospitals were absent from the 2023 AHA Annual Survey data. Missingness was associated with some observed characteristics, including educational attainment, practice setting, and Magnet status, but not with age, sex, employment status, moral distress, or either leadership responsiveness measure. These patterns supported the plausibility of the missing‐at‐random (MAR) assumption conditional on observed data.

Because the analytic sample was restricted to nurses with complete data on moral distress and the leadership responsiveness measures, missingness in the analytic models was confined to exogenous nurse‐ and hospital‐level covariates. Missing covariates were handled using multiple imputation by chained equations (MICE) for multilevel data (Grund et al. 2018). Missing hospital‐level covariates were imputed at the hospital level, whereas missing nurse‐level covariates were imputed using multilevel models that accounted for the clustering of nurses within hospitals. This approach preserved the hierarchical structure of the data and the level at which each covariate was measured. As a sensitivity analysis, we compared estimates obtained using multiple imputation with those from complete‐case analyses. The direction, magnitude, and statistical significance of the associations were substantively similar across approaches. Therefore, results from the multiply imputed analyses are reported.

## 3 Results

### 3.1 Differences in Moral Distress and Management Responsiveness by Magnet Status

Table 1 presents levels of moral distress, management responsiveness to patient care concerns, and administrative responsiveness to nurse concerns among nurses by Magnet status. Nurses in Magnet hospitals reported lower levels of moral distress than nurses in non‐Magnet hospitals (*M* = 2.41, SD = 0.86 vs. *M* = 2.46, SD = 0.87, *p* < 0.001). Nurses in Magnet hospitals also reported more favorable management responsiveness to patient care concerns compared with nurses in non‐Magnet hospitals (*M* = 2.19, SD = 1.02 vs. *M* = 1.99, SD = 0.96, *p* < 0.001). Similarly, nurses in Magnet hospitals reported more favorable administrative responsiveness to nurse concerns than nurses in non‐Magnet hospitals (*M* = 2.42, SD = 0.97 vs. *M* = 2.26, SD = 0.98, *p* < 0.001).

**TABLE 1:** Nurse Moral Distress, Leadership Responsiveness, and Characteristics by Hospital Magnet Status (*N* = 18,678).

Unadjusted nurse‐reported measures	Nurses in Magnet hospitals (*n* = 6170)	Nurses in Non‐Magnet hospitals (*n* = 12,508)	
	Mean (SD)	Mean (SD)	*p* ^e^
Nurse moral distress^a^	2.41 (0.86)	2.46 (0.87)	< 0.001
Leadership responsiveness			
Management responsiveness to patient care concerns^b^	2.19 (1.02)	1.99 (0.96)	< 0.001
Administration responsiveness to nurse concerns^c^	2.42 (0.97)	2.26 (0.98)	< 0.001

Nurse characteristics	Mean (SD)	Mean (SD)	*p* ^e^
Age	44.5 (12.9)	46.2 (12.5)	< 0.001

	*n* (%)	*n* (%)	*p* ^f^
Highest degree^d^			< 0.001
Baccalaureate or higher	5052 (84.1)	8437 (69.5)	
Less than a bachelor's degree	956 (15.9)	3695 (30.5)	
Sex^d^			< 0.001
Female	5222 (88.3)	10,365 (86.6)	
Male	691 (11.7)	1607 (13.4)	
Employment status^d^			0.569
Full‐time	4592 (74.5)	9364 (74.9)	
Part‐time	1569 (25.5)	3133 (25.1)	
Practice setting			0.619
ICU or psychiatric unit	1388 (22.5)	2772 (22.2)	
Settings other than ICU or psychiatric unit	4782 (77.5)	9736 (77.8)	

Abbreviations: ICU, intensive care unit; SD, standard deviation.

^a^ Values range from 1 to 4, with higher values indicating greater moral distress.

^b^ Values range from 1 to 4 and are based on the survey item, “How confident are you that management will act to resolve problems in patient care that nurses identify?” Higher values indicate more favorable management responsiveness to patient care concerns.

^c^ Values range from 1 to 4 and are based on the survey item, “Administration that listens and responds to nurse concerns.” Higher values indicate more favorable administration responsiveness to nurse concerns.

^d^ The numbers may not add up to the total because of missing values.

^e^ *p* values were calculated using two‐sample *t*‐tests.

^f^ *p* values were calculated using chi‐square tests.

### 3.2 Nurse and Hospital Characteristics by Magnet Status

Table 1 presents nurse characteristics by Magnet status. Among the 18,678 nurses, those working in Magnet hospitals were younger on average than those in non‐Magnet hospitals (*M* = 44.5, SD = 12.9 vs. *M* = 46.2, SD = 12.5, *p* < 0.001), were more likely to hold a baccalaureate degree or higher (84.1% vs. 69.5%, *p* < 0.001), and were more likely to be female (88.3% vs. 86.6%, *p* < 0.001).

Table 2 presents hospital characteristics by Magnet status. Among the 1829 hospitals, Magnet hospitals were more likely than non‐Magnet hospitals to be larger hospitals with more than 250 beds (70.0% vs. 24.6%, *p* < 0.001), teaching hospitals (64.6% vs. 54.7%, *p* = 0.007), not‐for‐profit institutions (99.6% vs. 75.4%, *p* < 0.001), and hospitals with high specialized service capacity (60.1% vs. 17.3%, *p* < 0.001).

**TABLE 2:** Hospital Characteristics (*N* = 1829).

Hospital characteristics	Magnet hospitals (*n* = 264)	Non‐Magnet hospitals (*n* = 1565)	*p* ^c^
	*n* (%)	*n* (%)	
Hospital size^a^			< 0.001
250 beds or fewer	67 (30.0)	970 (75.4)	
More than 250 beds	156 (70.0)	316 (24.6)	
Teaching status^a^			0.007
Non‐teaching	79 (35.4)	583 (45.3)	
Teaching	144 (64.6)	703 (54.7)	
Specialized service capacity^a^			< 0.001
High^b^	134 (60.1)	223 (17.3)	
Low	89 (39.9)	1063 (82.7)	
Ownership^a^			< 0.001
Not‐for‐profit	222 (99.6)	970 (75.4)	
For‐profit	1 (0.4)	316 (24.6)	

^a^ The numbers may not add up to the total because of missing values.

^b^ This category includes hospitals that provide advanced tertiary services (i.e., open‐heart surgery or major organ transplantation).

^c^ *p* values were calculated using chi‐square tests, except for ownership, for which Fisher's exact test was used because of small expected cell counts.

### 3.3 Multilevel Mediation Analyses

Table 3 presents the between‐ and within‐hospital associations among Magnet status, leadership responsiveness, and nurse moral distress. Intraclass correlation coefficient (ICC) estimates indicated that 4.4% of the variance in moral distress and 9.2% of the variance in management responsiveness to patient care concerns occurred between hospitals. In the administrative responsiveness model, 4.4% of the variance in moral distress and 8.4% of the variance in administrative responsiveness to nurse concerns occurred between hospitals.

**TABLE 3:** Associations Between Magnet Status, Leadership Responsiveness, and Nurse Moral Distress (*N* = 18,678).

Association	*b*	*β*	SE	95% CI	*p*
**Management responsiveness to patient care concerns**					
Between‐hospital associations					
Magnet → Management responsiveness (*a*_B_)	0.18	0.21	0.02	0.15 to 0.21	< 0.001
Management responsiveness → Moral distress (*b*_B_)	−0.31	−0.51	0.04	−0.39 to −0.23	< 0.001
Magnet → Moral distress (*c'*_B_)	−0.05	−0.09	0.02	−0.08 to −0.01	0.011
Within‐hospital association					
Management responsiveness → Moral distress (*b*_W_)	−0.34	−0.38	0.01	−0.35 to −0.33	< 0.001
Indirect association (*a*_B_ × *b*_B_)	−0.06	−0.11	0.01	−0.08 to −0.04	< 0.001
Total association (*c'*_B_ + *a*_B_ × *b*_B_)	−0.10	−0.19	0.02	−0.14 to −0.06	< 0.001
**Administration responsiveness to nurse concerns**					
Between‐hospital associations					
Magnet → Administration responsiveness (*a*_B_)	0.16	0.21	0.02	0.13 to 0.19	< 0.001
Administration responsiveness → Moral distress (*b*_B_)	−0.33	−0.51	0.04	−0.40 to −0.25	< 0.001
Magnet → Moral distress (*c'*_B_)	−0.05	−0.10	0.02	−0.09 to −0.02	0.004
Within‐hospital association					
Administration responsiveness → Moral distress (*b*_W_)	−0.30	−0.33	0.01	−0.31 to −0.29	< 0.001
Indirect association (*a*_B_ × *b*_B_)	−0.05	−0.10	0.01	−0.07 to −0.04	< 0.001
Total association (*c'*_B_ + *a*_B_ × *b*_B_)	−0.10	−0.19	0.02	−0.14 to −0.06	< 0.001

*Note:* *a*_B_ represents the between‐hospital association between Magnet status and leadership responsiveness; *b*_B_ represents the between‐hospital association between leadership responsiveness and moral distress; *b*_W_ represents the within‐hospital association between leadership responsiveness and moral distress; and *c'*_B_ represents the direct between‐hospital association between Magnet status and moral distress. The indirect association was calculated as *a*_B_ × *b*_B_. Monte Carlo 95% CIs were estimated for the indirect associations; 95% CIs for all other associations were based on the model estimates. Models accounted for the clustering of nurses within hospitals. Nurse characteristics (age, sex, educational attainment, employment status, and practice setting) were decomposed into within‐hospital and between‐hospital components, with group‐mean‐centered characteristics included at Level 1 and corresponding hospital means or proportions included at Level 2. Models additionally adjusted for hospital‐level teaching status, hospital size, specialized service capacity, and ownership.

Abbreviations: b, unstandardized coefficient; *β*, standardized coefficient; CI, confidence interval; SE, standard error.

#### 3.3.1 Management Responsiveness to Patient Care Concerns

At the between‐hospital level, Magnet status was associated with higher management responsiveness to patient care concerns (*a*_B_: unstandardized coefficient *b* = 0.18, 95% CI = 0.15 to 0.21, *p* < 0.001). Higher between‐hospital management responsiveness was associated with lower moral distress (*b*_B_: *b* = −0.31, 95% CI = −0.39 to −0.23, *p* < 0.001). Greater management responsiveness was also associated with lower moral distress at the within‐hospital level (*b*_W_: *b* = −0.34, 95% CI = −0.35 to −0.33, *p* < 0.001).

The between‐hospital indirect association between Magnet status and moral distress involving management responsiveness (*a*_B_ × *b*_B_) was significant (*b* = −0.06, Monte Carlo 95% CI = −0.08 to −0.04, *p* < 0.001). The direct between‐hospital association between Magnet status and moral distress remained significant after accounting for management responsiveness (*c'*_B_: *b* = −0.05, 95% CI = −0.08 to −0.01, *p* = 0.011). The total association was also significant (*b* = −0.10, 95% CI = −0.14 to −0.06, *p* < 0.001).

#### 3.3.2 Administrative Responsiveness to Nurse Concerns

The analysis using administrative responsiveness to nurse concerns yielded similar findings. At the between‐hospital level, Magnet status was associated with higher administrative responsiveness (*a*_B_: *b* = 0.16, 95% CI = 0.13 to 0.19, *p* < 0.001), and higher administrative responsiveness was associated with lower moral distress (*b*_B_: *b* = −0.33, 95% CI = −0.40 to −0.25, *p* < 0.001). Greater administrative responsiveness was also associated with lower moral distress at the within‐hospital level (*b*_W_: *b* = −0.30, 95% CI = −0.31 to −0.29, *p* < 0.001).

The between‐hospital indirect association between Magnet status and moral distress involving administrative responsiveness (*a*_B_ × *b*_B_) was significant (*b* = −0.05, Monte Carlo 95% CI = −0.07 to −0.04, *p* < 0.001). The direct between‐hospital association between Magnet status and moral distress remained significant after accounting for administrative responsiveness (*c'*_B_: *b* = −0.05, 95% CI = −0.09 to −0.02, *p* = 0.004). The total association was also significant (*b* = −0.10, 95% CI = −0.14 to −0.06, *p* < 0.001).

## 4 Discussion

In this large multistate study of almost 20,000 direct care nurses, we found that nurses working in Magnet hospitals reported lower levels of moral distress and more favorable assessments of management responsiveness than nurses working in non‐Magnet hospitals. Greater management responsiveness was also associated with lower levels of moral distress at both the within‐ and between‐hospital levels. Multilevel mediation analyses demonstrated a significant between‐hospital indirect association between Magnet status and moral distress involving management responsiveness, suggesting that management responsiveness may account for part of the observed association between Magnet status and moral distress. Similar findings were observed in the analysis using a related measure of administrative responsiveness to nurse concerns, providing additional support for the consistency of the findings across the two leadership responsiveness measures.

Our findings contribute to the growing literature on moral distress by highlighting the importance of organizational factors, particularly leadership responsiveness, in nurses' experiences of moral distress (Bolton et al. 2025; Booth and Robinson 2025). Previous research has primarily focused on nurse‐, patient‐, and ethical climate‐related factors, such as nurses' personal characteristics, years of experience, and patient acuity (Atashzadeh‐Shoorideh et al. 2021; Lamoureux et al. 2024). Similarly, interventions designed to address moral distress have largely targeted individual nurses through approaches such as moral empowerment programs, end‐of‐life education, reflective writing, group debriefings, multidisciplinary case discussions, and moral resilience training (Imbulana et al. 2021). Our findings suggest that nurses who report more favorable assessments of management responsiveness to patient care concerns also report lower levels of moral distress. As frontline observers of patient needs and safety concerns, nurses may feel better supported in fulfilling their professional and ethical responsibilities when they perceive that organizational leaders respond effectively to the concerns they raise (Bolton et al. 2025).

The association between management responsiveness and moral distress was evident at both the within‐ and between‐hospital levels. Within hospitals, nurses who perceived management as more responsive relative to other nurses in the same hospital reported lower moral distress. At the between‐hospital level, hospitals characterized by more favorable assessments of management responsiveness also had lower levels of moral distress. These findings suggest that leadership responsiveness may reflect both nurses' individual experiences with management and broader organizational differences across hospitals. Leadership responsiveness likely operates within the broader nursing practice environment, including factors such as staffing adequacy, interdisciplinary collaboration, and organizational support (Lake 2002). These work environment characteristics may be related to nurses' experiences of moral distress by shaping the organizational context in which nurses deliver care, advocate for patients, and raise concerns. Future longitudinal and intervention studies are needed to determine whether changes in leadership responsiveness are followed by changes in nurses' moral distress.

Several implications for nursing leadership emerge from these findings. Moral distress is often understood in relation to ethically challenging clinical situations. However, our findings suggest that the organizational context in which nurses raise and address patient care concerns may also be important. Nurse leaders and healthcare organizations may therefore consider strengthening mechanisms through which nurses can raise patient care concerns, participate in decision‐making, and receive timely and consistent responses from leadership (Bolton et al. 2025). Leadership development initiatives emphasizing communication, advocacy, accountability, and responsiveness may also warrant further evaluation as potential organizational strategies for addressing moral distress.

Consistent with the principles of the Magnet Model (American Nurses Credentialing Center 2025) and prior Magnet research demonstrating more favorable patient and nurse outcomes (Yu, Aiken, et al. 2026; Yu, Brom, et al. 2026), including lower burnout and intent to leave, nurses working in Magnet hospitals reported lower levels of moral distress and more favorable assessments of management responsiveness than nurses working in non‐Magnet hospitals. Magnet hospitals emphasize and invest in leadership development, shared governance, structural empowerment, and organizational processes (Yu, Keele, et al. 2026) that encourage nurses to participate in decision‐making and raise concerns regarding patient care. These organizational characteristics may foster greater confidence among nurses that their concerns will be heard and addressed, which may be associated with lower levels of moral distress. Lower levels of moral distress may also be associated with the more favorable job outcomes consistently observed among nurses in Magnet hospitals, given the well‐established associations between moral distress, burnout, job dissatisfaction, and turnover intentions (Eche et al. 2023; Karakachian and Colbert 2019). Future research should further examine moral distress as a potential mechanism linking supportive organizational environments with nurse workforce outcomes and evaluate interventions designed to strengthen leadership responsiveness and nurses' ability to advocate for patient care concerns.

## 5 Limitations

This study has several limitations. First, the cross‐sectional design precludes causal inference and does not establish temporal ordering among Magnet status, management responsiveness, and moral distress. Although the multilevel mediation model was informed by the SPO framework, the indirect associations should be interpreted as statistical associations consistent with the proposed conceptual model rather than evidence of a causal mediation mechanism. Second, although the sample included nurses from 10 US states and a large number of hospitals, the findings may not be generalizable to all healthcare settings or international contexts. Third, moral distress and leadership responsiveness were assessed using single‐item measures, which may not fully capture the multidimensional nature of these constructs and may be subject to measurement error. In addition, both measures were reported by the same nurses, raising the possibility of common‐method bias that could contribute to the observed associations. Finally, because both measures were assessed concurrently, the direction of their association cannot be established. For example, nurses experiencing greater moral distress may assess leadership responsiveness less favorably. Future longitudinal studies using validated multi‐item measures and data from multiple sources are needed to further examine these relationships. Despite these limitations, this study has several strengths, including a large multistate sample of nurses, the use of MSEM to account for clustering and distinguish between within‐ and between‐hospital associations, and the examination of two conceptually related measures of management responsiveness. Together, these strengths provide a rigorous assessment of organizational factors associated with nurses' experiences of moral distress.

## 6 Conclusions

Nurses working in Magnet hospitals reported lower levels of moral distress and more favorable assessments of management responsiveness to patient care concerns than nurses working in non‐Magnet hospitals. Greater management responsiveness was associated with lower moral distress and accounted for part of the association between Magnet status and moral distress. These findings suggest that leadership responsiveness to nurses' patient care concerns may represent an important and potentially modifiable organizational factor associated with nurses' experiences of moral distress. Efforts to strengthen management responsiveness, amplify nurses' voices, and create supportive practice environments warrant further evaluation as potential organizational strategies for addressing moral distress and supporting nurse well‐being.

## 7 Clinical Resources

American Nurses Credentialing Center. Magnet Model—Creating a Magnet Culture. https://www.nursingworld.org/organizational‐programs/magnet/magnet‐model/. American Association of Critical‐Care Nurses. Moral Distress in Nursing: What You Need to Know. https://www.aacn.org/clinical‐resources/moral‐distress. American Nurses Association. Leadership in Nursing: Qualities & Why It Matters. https://www.nursingworld.org/content‐hub/resources/nursing‐leadership/leadership‐in‐nursing/.

## Funding

This work was supported by the National Institute of Nursing Research, R01NR014855, T32NR007104. National Council of State Boards of Nursing.

## Conflicts of Interest

The authors declare no conflicts of interest.

## Data Availability

Research data are not shared.
